# Factors associated with uptake of home-based HIV counselling and testing and HIV care services among identified HIV-positive persons in Masaka, Uganda

**DOI:** 10.1080/09540121.2018.1441967

**Published:** 2018-02-20

**Authors:** Eugene Ruzagira, Kathy Baisley, Anatoli Kamali, Heiner Grosskurth

**Affiliations:** a Department Infectious Disease and Epidemiology, London School of Hygiene and Tropical Medicine, London, United Kingdom; b MRC/UVRI Uganda Research Unit on AIDS, Entebbe, Uganda; c International AIDS Vaccine Initiative, New York, USA

**Keywords:** HIV/AIDS, HIV counselling and testing, linkage to HIV care, Uganda, Africa

## Abstract

We investigated uptake of home-based HIV counselling and testing (HBHCT) and HIV care services post-HBHCT in order to inform the design of future HBHCT programmes. We used data from an open-label cluster-randomised controlled trial which had demonstrated the effectiveness of a post-HBHCT counselling intervention in increasing linkage to HIV care. HBHCT was offered to adults (≥18 years) from 28 rural communities in Masaka, Uganda; consenting HIV-positive care naïve individuals were enrolled and referred for care. The trial's primary outcome was linkage to HIV care (clinic-verified registration for care) six months post-HBHCT. Random effects logistic regression was used to investigate factors associated with HBHCT uptake, linkage to care, CD4 count receipt, and antiretroviral therapy (ART) initiation; all analyses of uptake of post-HBHCT services were adjusted for trial arm allocation. Of 13,455 adults offered HBHCT, 12,100 (89.9%) accepted. HBHCT uptake was higher among men [adjusted odds ratio (aOR) 1.20, 95% confidence interval (CI) = 1.07–1.36] than women, and decreased with increasing age. Of 551 (4.6%) persons who tested HIV-positive, 205 (37.2%) were in care. Of those not in care, 302 (87.3%) were enrolled in the trial and of these, 42.1% linked to care, 35.4% received CD4 counts, and 29.8% initiated ART at 6 months post-HBHCT. None of the investigated factors was associated with linkage to care. CD4 count receipt was lower in individuals who lived ≥30 min from an HIV clinic (aOR 0.60, 95%CI = 0.34–1.06) versus those who lived closer. ART initiation was higher in older individuals (≥45 years versus <25 years, aOR 2.14, 95% CI = 0.98–4.65), and lower in single (aOR 0.60, 95% CI = 0.28–1.31) or divorced/separated/widowed (aOR 0.47, 95% CI = 0.23–0.93) individuals versus those married/cohabiting. HBHCT was highly acceptable but uptake of post-HBHCT care was low. Other than post-HBHCT counselling, this study did not identify specific issues that require addressing to further improve linkage to care.

## Introduction

HIV counselling and testing (HCT) is essential for expanding HIV prevention and treatment services (Matovu & Makumbi, 2007). Although access to HCT in sub-Saharan Africa (SSA) has increased significantly, its uptake remains low (WHO, 2015). For instance, only 60% of HIV-positive adults know their HIV status (UNAIDS, 2016). Consequently, many HIV-positive people present late for care (Siedner et al., 2015) and AIDS-related morbidity and mortality remain high (UNAIDS, 2014). In order to expand HCT coverage, WHO recommends the use of facility-based and community-based HCT models (WHO, 2015).

Home-based HIV testing and counselling (HBHCT) is a highly acceptable community-based HCT model that has the potential to substantially increase knowledge of HIV status in SSA (Sabapathy et al., 2012). However, data on linkage to HIV care facilities (Medley et al., 2013) or the uptake of specific HIV care services (Sharma, Ying, Tarr, & Barnabas, 2015) after HBHCT are limited. These data suggest that in the absence of interventions to facilitate linkage, up to 70% of HIV-positive persons identified through HBHCT in SSA do not link to an HIV clinic, and that an even higher proportion do not take up specific HIV care services (Ruzagira, Baisley, Kamali, Biraro, & Grosskurth, 2017). In order to design effective HBHCT programmes, there is a need to understand the determinants of HBHCT uptake and linkage to and uptake of HIV care services after HBHCT.

## Methods

### Setting and study population

Data used for this analysis were collected during an open-label cluster randomised controlled trial that evaluated the effectiveness of a counselling intervention on linkage to HIV care between March 2015 and March 2016 in Masaka district, Uganda (Ruzagira, Baisley, Kamali, & Grosskurth, 2017). Twenty-eight rural communities (clusters) were randomly allocated to intervention (HBHCT, care referral and follow-up counselling) or control (HBHCT and care referral only) arms (*n* = 14 clusters/arm). Randomisation was stratified on distance from the district capital (≤10 vs >10 km) and cluster composition (larger single village vs combined small villages), and restricted to achieve balance on the following cluster-level covariates: cluster size; presence of a trading centre; location along a major road; lakeshore location; and presence of an HIV clinic within 5 km. HBHCT was offered to all adults (≥18 years) in the randomised communities. Identified HIV-positive individuals who were not previously or currently in HIV care, available for follow-up, and not participating in other health-related research were eligible for the trial.

### Study outcomes

The primary outcome for the HBHCT component of the study was uptake of HBHCT. The trial primary outcomes were linkage to care (clinic-verified registration for HIV care) determined six months after HIV diagnosis, and time to linkage. As reported previously, the trial intervention significantly increased linkage to HIV care and uptake of HIV care services (Ruzagira, Grosskurth, Kamali, & Baisley, 2017). This paper investigates factors associated with HBHCT uptake (i.e., the proportion who consented to an HIV test and received the result at home) among persons who were offered HBHCT. We also investigate factors associated with linkage to care (i.e., the proportion with confirmed registration at an HIV clinic), CD4 count receipt (i.e., the proportion who received a CD4 count), and ART initiation (i.e., the proportion who initiated ART) among individuals who were enrolled in the trial.

### Study procedures

#### HBHCT

Community residents were informed about HBHCT activities through village meetings and/or door-to-door mobilisation by study staff and village health team members. Three to seven days later, study counsellors visited all the households in a community, enumerated resident adults, and conducted HBHCT for those who consented. HIV testing was done using rapid test kits as reported elsewhere (Ruzagira, Grosskurth, Kamali, & Baisley, 2017). Age, sex, HBHCT uptake, and reasons for non-uptake, and HIV test result data were recorded on a questionnaire. Information on previous HCT and/or the results was not recorded except as a reason for declining HBHCT.

#### Referral for HIV care and enrolment into the trial

Trial procedures have been described previously (Ruzagira, Baisley, Kamali, Biraro, et al., 2017). Briefly, individuals who tested HIV-positive and reported that they had never received HIV care were given a referral letter to take to their preferred HIV clinic. Additionally, they were given information about the trial and invited to participate. Individuals who enrolled in the trial completed a counsellor-administered questionnaire to collect sociodemographic data and HCT history.

#### Follow-up counselling intervention

In intervention clusters, counsellors visited trial volunteers’ homes at one and two months post-HBHCT to provide counselling aiming to encourage linkage to HIV care.

#### Collection of HIV care outcomes

Six months post-HBHCT, counsellors visited trial volunteers and used a questionnaire to interview them about HIV care outcomes. For volunteers who reported that they had linked to care, a study team member visited the clinic to verify linkage and other HIV care outcomes. Volunteers whose records could not be found at the clinics were considered not to have linked to care.

### Data management

Questionnaires were checked for completeness before data entry. Data were double-entered and validated in Microsoft Access. Checks were run on the entered data and any generated queries resolved.

### Sample size

The trial was designed to have >80% power to detect an increase in linkage to care among trial volunteers, from 35% in the control arm to 60% in the intervention arm at a significance level of 5%. Details have been reported elsewhere (Ruzagira, Baisley, Kamali, & Grosskurth, 2017).

### Statistical analysis

Pearson chi-squared statistics with the second-order correction of Rao and Scott to account for the clustered design were used to examine differences between variables of interest. Random effects logistic regression models were used to investigate factors associated with each outcome, accounting for the clustered design. All analyses were conducted using Stata 12 (StataCorp, College Station, Texas, USA).

#### HBHCT

For the analysis of potential determinants of HBHCT uptake, no individual level variables, other than age and sex, were available. Age and sex were included a priori in all models. Cluster-level variables were then added one at a time and retained if associated with HBHCT uptake at *p *< 0.10.

#### HIV care outcomes

Potential determinants of each outcome were examined using a conceptual framework (Victora, Huttly, Fuchs, & Olinto, 1997) with three domains: cluster-level factors (as the most distal determinants), sociodemographic factors, and HCT history factors. Trial arm and stratum (trial design factors), and age and sex (potential confounders) were included a priori in all models. Cluster-level factors were added one by one to the trial design-, age- and sex-adjusted model and retained in a “core” model if they remained associated with the outcome under consideration at *p* < 0.10. Sociodemographic variables were then added one by one to the core model, and retained if they remained associated with the outcome under consideration at *p* < 0.10. Lastly, HCT history factors (as the most proximate determinants) were added and retained if they remained associated with the outcome under consideration at *p* < 0.10. Volunteers who had been lost to follow-up were assumed not to have linked to care or attained other outcomes.

### Ethical considerations

The trial was approved by the Uganda Virus Research Institute Ethics Committee, the Uganda National Council for Science and Technology, and the London School of Hygiene and Tropical Medicine Ethics Committee. Written informed consent was obtained from each individual.

## Results

### HBHCT uptake

A total of 15,096 adults were enumerated in the 28 clusters; over half were women (7946, 52.6%) and <35 years (7909, 52.4%). Of those enumerated, 13,455 (89.1%) were found at home and offered HBHCT. Absence from home was more common among men than women [1313 (18.4%) vs. 328 (4.1%); *p* < 0.001] and among those aged ≥25 years than those <25 years [1235 (11.4%) vs. 189 (4.8%); *p* < 0.001; age missing for 320 individuals]. Of those found at home, 12,100 (89.9%) accepted HBHCT. The median age of HBHCT acceptors was 31 years (women, 30 years; men; 32 years) (Table 1). HBHCT uptake was higher among men [adjusted odds ratio (aOR) 1.20, 95% confidence interval (CI) 1.07–1.36] than women, and decreased with increasing age. None of the cluster-level characteristics was associated with HBHCT uptake.

**Table 1.** Factors associated with uptake of HBHCT in Masaka, Uganda.

			Crude analysis		Adjusted analysis	
	*N*	Accepted HBHCT (%)	OR (95% CI)	*P*-value	aOR (95% CI)§	*P*-value
**Total**	13455	12100	–	–	–	–
**Age and sex**						
Median age in years (IQR)	32 (24–46)	31 (23–45)	–	–	–	–
Age group (years)						
18–24	3732	3595 (96.3)	1	<0.001	1	<0.001
25–34	3550	3258 (91.8)	0.42 (0.34–0.52)		0.42 (0.34–0.52)	
35–44	2419	2152 (89.0)	0.31 (0.25–0.38)		0.31 (0.25–0.38)	
45+	3651	3094 (84.7)	0.21 (0.17–0.26)		0.21 (0.17–0.26)	
Missing age	103	1 (<1.0)	–		–	–
Sex						
Female	7618	6827 (89.6)	1	0.13	1	0.003
Male	5837	5273 (90.3)	1.09 (0.97–1.23)		1.20 (1.07–1.36)	
**Cluster-level variables**					aOR (95% CI)*	
Distance from district capital (stratum)						
≥10 Km	7956	7106 (89.3)	1	0.23	1	0.41
<10 Km	5499	4994 (90.8)	1.17 (0.91–1.52)		1.10 (0.87–1.39)	
Presence of a trading centre						
No	6825	6095 (89.3)	1	0.43	1	0.39
Yes	6630	6005 (90.6)	1.11 (0.86–1.44)		1.11 (0.88–1.39)	
Located on lakeshore						
No	11079	9988 (90.2)	1	0.44	1	0.39
Yes	2376	2112 (88.9)	0.87 (0.60–1.24)		0.87 (0.63–1.20)	
HIV clinic within 5 km						
No	2995	2665 (90.0)	1	0.40	1	0.48
Yes	10460	9435 (90.2)	1.14 (0.84–1.56)		1.11 (0.84–1.46)	
Located along a major road						
No	9823	8870 (90.3)	1	0.43	1	0.20
Yes	3632	3,230 (88.9)	0.89 (0.66–1.19)		0.84 (0.65–1.09)	

§Age and sex included a priori; IQR, interquartile range; *Adjusted for age and sex.

Among the 1355 who declined HBHCT, prior knowledge of HIV-positive status and receipt of care was the most common reason (437, 32.3%) for declining; this was more common among women than men [287 (36.3%) vs. 150 (26.6%); *p* = 0.002] and among those aged ≥25 years than those <25 years [401 (35.9%) vs. 21 (15.3%); *p* < 0.001]. Other reasons were a lack of interest in HCT (400, 29.5%); having recently tested for HIV and not wanting a repeat test (214, 15.8%); low HIV risk perception (92, 6.8%); and inability to consent (8, 0.6%). Reasons were not documented for 204 (15.1%) individuals.

A total of 551 (4.6% of HBHCT acceptors) tested HIV-positive; of these, 308 (55.9%) were newly diagnosed, 205 (37.2%) had previously tested HIV-positive and were engaged in care, and 38 (6.9%) had previously tested HIV-positive but were not in care.

### Enrolment into the trial

Of the 346 (308 newly diagnosed; 38 previously diagnosed) HIV-positive individuals who were not in HIV care, 302 (87.3%) were enrolled in the trial (intervention arm, *n* = 149). The median age of those enrolled was 30.0 years (women, 29 years; men, 32 years); most had received prior HCT (80.5%) but only 12.3% were aware of their HIV-positive status (Table 2). Twenty-five (8.3%) individuals were lost to follow-up.

**Table 2.** Factors associated with linkage to HIV care after HBHCT in Masaka, Uganda.

	*N* (Column %)	Linked (Row %)	Crude analysis		Adjusted analysis	
			OR (95% CI)	*P*-value	aOR (95% CI)§	*P*-value
**Total**	302	127				
**Age and sex**						
Median age in years (IQR)	30 (25–39)	31 (26–42)	–	–	–	–
Age group (years)						
18–24	71 (23.5)	25 (35.2)	1	0.37	1	0.28
25–34	121 (40.1)	51 (42.2)	1.30 (0.69–2.45)		1.28 (0.68–2.40)	
35–44	59 (19.5)	24 (40.7)	1.16 (0.55–2.45)		1.23 (0.59–2.60)	
≥45	51 (16.9)	27 (52.9)	1.96 (0.91–4.22)		2.12 (0.99–4.55)	
Sex						
Female	165 (54.6)	72 (43.6)	1	0.51	1	0.44
Male	137 (45.4)	55 (40.2)	0.85 (0.53–1.38)		0.83 (0.51–1.34)	
**Cluster-level factors**					aOR (95% CI)*	
Presence of a trading centre						0.92
No	177 (58.6)	72 (40.7)	1	0.73	0.97 (0.54–1.73)	
Yes	125 (41.4)	55 (44.0)	1.12 (0.60–2.09)			
Located on lakeshore						
No	237 (78.5)	100 (42.2)	1	0.95	1	0.90
Yes	65 (21.5)	27 (41.5)	1.03 (0.45–2.33)		0.95 (0.46–1.96)	
HIV clinic within 5 km						
No	80 (26.5)	31 (38.8)	1	0.69	1	0.75
Yes	222 (73.5)	96 (43.2)	1.16 (0.56–2.41)		0.90 (0.45–1.78)	
Located along a major road						
No	216 (71.5)	87 (40.3)	1	0.46	1	0.47
Yes	86 (28.5)	40 (46.5)	1.30 (0.65–2.59)		1.26 (0.67–2.36)	
**Individual-level sociodemographic factors**					aOR (95% CI)*	
Marital status						
Married/cohabiting	182 (60.3)	75 (41.2)	1	0.41	1	0.86
Single	48 (15.9)	17 (35.4)	0.79 (0.39–1.58)		0.84 (0.42–1.70)	
Divorced/separated/widowed	72 (23.8)	35 (48.6)	1.33 (0.74–2.37)		1.05 (0.57–1.93)	
Education						
None/incomplete primary	182 (60.3)	78 (42.9)	1	0.94	1	0.86
Primary	66 (21.9)	26 (39.4)	0.90 (0.49–1.66)		0.84 (0.46–1.56)	
Above primary	54 (17.9)	23 (42.6)	0.93 (0.49–1.78)		0.92 (0.48–1.77)	
Socio-economic status						
Low	117 (38.7)	50 (42.7)	1	0.91	1	0.93
Middle	108 (35.8)	46 (42.6)	1.01 (0.58–1.77)		1.04 (0.60–1.81)	
High	77 (25.5)	31 (40.3)	0.89 (0.48–1.66)		0.92 (0.49–1.72)	
Religion						
Catholic	213 (70.5)	88 (41.3)	1	0.17	1	0.20
Anglican	42 (13.9)	22 (52.4)	1.55 (0.76–3.15)		1.45 (0.71–2.96)	
Muslim	34 (11.3)	10 (29.4)	0.54 (0.23–1.26)		0.53 (0.23–1.22)	
Other	13 (4.3)	7 (53.9)	1.70 (0.52–5.50		1.48 (0.45–4.83)	
Time to nearest HIV clinic						
<30 min	86 (28.5)	44 (51.2)	1	0.05	1	0.11
≥30 min	216 (71.5)	83 (38.4)	0.58 (0.33–0.99)		0.64 (0.36–1.11)	
**HCT history factors**					aOR (95% CI)*	
Ever tested for HIV						
No	59 (19.5)	24 (40.7)	1	0.75	1	0.87
Yes	243 (80.5)	103 (42.4)	1.10 (0.60–2.02)		1.05 (0.57–1.95)	
Aware of HIV-positive status						
No	265 (87.7)	112 (42.3)	1	0.91	1	0.87
Yes	37 (12.3)	15 (40.5)	0.96 (0.46–2.00)		0.94 (0.45–1.96)	

§Adjusted for trial arm and stratum; IQR, interquartile range; *Adjusted for trial arm, stratum, age and sex.

### Linkage to HIV care

Overall, 127 (42.1%) of the individuals enrolled in the trial linked to HIV care. Linkage was lower among individuals who required ≥30 min to travel to the nearest HIV clinic than those who required less time, but there was no evidence of a significant difference (aOR 0.64, 95% CI 0.36–1.11) (Table 2). No other characteristics were associated with linkage to care.

### Receipt of CD4 counts

Overall, 107 (35.4%) of the individuals enrolled in the trial received a CD4 count. There was some evidence that individuals who required ≥30 min of travel time to the nearest HIV clinic were less likely (aOR 0.60, 95% CI 0.34–1.06) to receive CD4 counts than those who required less time (Table 3). No other characteristics were associated with CD4 count receipt.

**Table 3.** Factors associated with obtaining CD4 counts and initiation of ART after HBHCT in Masaka, Uganda.

	Obtaining CD4 counts			Initiating ART		
	*N* (%)	Crude analysis	Adjusted analysis	*N* (%)	Crude analysis	Adjusted analysis
		OR (95% CI)	aOR (95% CI)§		OR (95% CI)	aOR (95% CI)§
**Age and sex**						
Age group (years)		*P* = 0.30	*P* = 0.21		*P* = 0.10	*P* = 0.10
18–24	21 (29.6)	1	1	19 (27.8)	1	1
25–34	42 (34.7)	1.22 (0.63–2.37)	1.21 (0.63–2.32)	36 (29.8)	1.14 (0.59–2.23)	1.11 (0.57–2.17)
35–44	20 (33.9)	1.11 (0.51–2.42)	1.23 (0.57–2.69)	13 (22.0)	0.75 (0.33–1.71)	0.78 (0.34–1.79)
≥45	24 (47.1)	2.04 (0.94–4.47)	2.24 (1.03–4.88)	22 (43.1)	2.09 (0.96–4.55)	2.14 (0.98–4.65)
Sex		*P* = 0.74	*P* = 0.66		*P* = 0.36	*P* = 0.39
Female	60 (36.4)	1	1	53 (32.1)	1	1
Male	47 (34.3)	0.92 (0.56–1.51)	0.89 (0.54–1.47)	37 (27.0)	0.79 (0.48–1.31)	0.80 (0.48–1.33)
**Cluster-level factors**			aOR (95% CI)*			aOR (95% CI)*
Presence of a trading centre		*P* = 0.93	*P* = 0.70		*P* = 0.55	*P* = 0.43
No	62 (35.0)	1	1	50 (28.3)	1	1
Yes	45 (36.0)	1.03 (0.54–1.95)	0.90 (0.51–1.58)	40 (32.0)	1.18 (0.68–2.06)	1.26 (0.72–2.19)
Located on lakeshore		*P* = 0.83	*P* = 0.76		*P* = 0.58	*P* = 0.66
No	85 (35.9)	1	1	73 (30.8)	1	1
Yes	22 (33.9)	0.91 (0.40–2.10)	0.90 (0.45–1.79)	17 (26.2)	0.82 (0.41–1.65)	0.85 (0.41–1.76)
HIV clinic within 5 km		*P* = 0.43	*P* = 0.88		*P* = 0.74	*P* = 0.74
No	25 (31.3)	1	1	22 (27.5)	1	1
Yes	82 (36.9)	1.36 (0.64–2.89)	0.95 (0.50–1.81)	68 (30.6)	1.12 (0.59–2.14)	0.89 (0.43–1.81)
Located along a major road		*P* = 0.34	*P* = 0.56		*P* = 0.18	*P* = 0.34
No	72 (33.3)	1	1	59 (27.3)	1	1
Yes	35 (40.7)	1.40 (0.70–2.82)	1.25 (0.68–2.31)	31 (36.1)	1.49 (0.84–2.64)	1.36 (0.73–2.54)
**Individual-level sociodemographic factors**			aOR (95% CI)*			aOR (95% CI)*
Marital status		*P* = 0.59	*P* = 0.87		*P* = 0.19	*P* = 0.06
Married/cohabiting	60 (33.0)	1	1	61 (33.5)	1	1
Single	17 (35.4)	1.12 (0.55–2.25)	1.20 (0.59–2.43)	11 (22.9)	0.57 (0.27–1.23)	0.60 (0.28–1.31)
Divorced/separated/widowed	30 (41.7)	1.37 (0.75–2.47)	1.08 (0.58–2.01)	18 (25.0)	0.64 (0.34–1.21)	0.47 (0.23–0.93)
Education		*P* = 0.98	*P* = 0.90		*P* = 0.87	*P* = 0.74
None/incomplete primary	65 (35.7)	1	1	56 (30.8)	1	1
Primary	22 (33.3)	0.95 (0.50–1.78)	0.86 (0.46–1.62)	18 (27.3)	0.85 (0.45–1.61)	0.78 (0.40–1.49)
Above primary	20 (37.0)	1.00 (0.52–1.94)	0.99 (0.51–1.93)	16 (29.6)	0.92 (0.47–1.82)	0.89 (0.45–1.78)
Socio-economic status		*P* = 0.98	*P* = 0.97		*P* = 0.70	*P* = 0.80
Low	41 (35.0)	1	1	38 (32.5)	1	1
Middle	38 (35.2)	1.04 (0.58–1.85)	1.06 (0.60–1.87)	30 (27.8)	0.80 (0.44–1.43)	0.83 (0.46–1.50)
High	28 (36.4)	1.07 (0.57–2.02)	1.07 (0.57–2.03)	42 (28.6)	0.81 (0.42–1.54)	0.84 (0.44–1.63)
Religion		*P* = 0.33	*P* = 0.39		*P* = 0.06	*P* = 0.11
Catholic	74 (34.7)	1	1	57 (26.8)	1	1
Anglican	19 (45.2)	1.59 (0.78–3.27)	1.40 (0.68–2.89)	19 (45.2)	2.26 (1.12–4.54)	2.03 (0.99–4.16)
Muslim	9 (26.5)	0.62 (0.26–1.48)	0.57 (0.24–1.37)	6 (23.5)	0.82 (0.35–1.96)	0.73 (0.30–1.78)
Other	5 (38.5)	1.23 (0.36–4.15)	0.95 (0.28–3.26)	8 (46.2)	2.42 (0.76–7.71)	2.17 (0.66–7.11)
Time to nearest HIV clinic		*P* = 0.03	*P* = 0.08		*P* = 0.58	*P* = 0.81
<30 min	39 (45.4)	1	1	28 (32.6)	1	1
≥30 min	68 (31.5)	0.53 (0.30–0.92)	0.60 (0.34–1.06)	62 (28.7)	0.85 (0.49–1.49)	0.93 (0.53–1.65)
**HCT history factors**			aOR (95% CI)†			aOR (95% CI)†
Ever tested for HIV		*P* = 0.80	*P* = 0.61		*P* = 0.89	*P* = 0.51
No	22 (37.3)	1	1	18 (30.5)	1	1
Yes	85 (35.0)	0.92 (0.50–1.71)	0.85 (0.45–1.60)	72 (29.6)	0.96 (0.51–1.79)	0.80 (0.41–1.56)
Aware of HIV-positive status		*P* = 0.96	*P* = 0.99		*P* = 0.22	*P* = 0.31
No	94 (35.5)	1	1	76 (28.7)	1	1
Yes	13 (35.1)	1.02 (0.48–2.17)	0.99 (0.46–2.14)	14 (37.8)	1.60 (0.76–3.36)	1.48 (0.69–3.18)

§Adjusted for trial arm and stratum; *Adjusted for trial arm, stratum, age and sex; †Adjusted for trial arm, stratum, age, sex and time to nearest clinic (for obtaining CD4 count outcome), or marital status (for ART initiation outcome).

### Initiation of ART

Of the individuals enrolled in the trial, 90 (29.8%) initiated ART. This included 61 (95.3%) of 64 individuals who had a CD4 count of ≤500 cells/mm^3^, the ART eligibility threshold at the time of the study (Uganda Ministry of Health, 2014). There was some evidence that ART initiation was associated with age, with individuals aged ≥45 years most likely to initiate ART (aOR 2.14, 95% CI 0.98–4.65, compared with those <25 years) (Table 3). Older individuals (≥45 years) were more likely to have a CD4 count of ≤500 cells/mm^3^ than those who were younger [20 (83.3%) vs. 44 (53.0%), *p* < 0.001] at the time of linkage to care. There was also weak evidence of an association with marital status, with single (aOR 0.60, 95% CI 0.28–1.31) and divorced/separated/widowed (aOR 0.47, 95% CI 0.23–0.93) individuals less likely to initiate ART than married/cohabiting individuals. No other characteristics were associated with ART initiation.

## Discussion

We observed high (>80%) levels of HBHCT uptake in this rural population. These findings are consistent with those from other settings (Sharma et al., 2015), and reaffirm the potential of HBHCT to complement other HCT approaches in SSA (Sabapathy et al., 2012). Although more women than men were found at home in this study, men were more likely than women to accept HBHCT. A possible reason for this finding may be that compared to men, women are more likely to attend health care facilities and consequently to learn their HIV status through facility-based HCT programmes (Sekandi et al., 2011).

Similar to other studies (Dalal et al., 2013; Helleringer, Kohler, Frimpong, & Mkandawire, 2009), HBHCT uptake was highest in the youngest age group. Possible reasons for this include a lower HIV risk perception in older compared to younger persons (Sekandi et al., 2011) and the perception of HBHCT as “youth friendly” (Jürgensen et al., 2013). The HBHCT model is well-suited to young people's HIV testing delivery preferences. These include the need for compassionate, friendly, and competent staff; counselling services provided alongside the testing; and testing which is both rapid and free (WHO, 2013). In the context of our study, an additional explanation may be that older persons are more likely than younger ones to be already aware of their HIV status. We did not collect data on previous HIV testing at the time of HBHCT, except as a reason for declining HBHCT; therefore, we could not examine if older age was associated with prior knowledge of HIV status among all individuals. However, our finding that knowledge of HIV-positive status was a more common reason for declining HBHCT among persons aged ≥25 years than those <25 years suggests that this may be the case.

Consistent with findings from other studies (Sharma et al., 2015), the proportion of individuals who tested HIV-positive out of those who accepted HBHCT was relatively low. This finding implies that large numbers of people may need to be tested to identify HIV-positive persons, and that in low HIV prevalence populations, HBHCT may not be not feasible. This limitation should however be weighed against the potential of HBHCT to reach previously untested persons compared to other HCT strategies (Menzies et al., 2009) and identify previously undiagnosed HIV infection (Sabapathy et al., 2012). Indeed, over 50% of those who tested HIV-positive in our study were newly diagnosed.

Despite a high HBHCT uptake, overall linkage to HIV care among HIV-positive care naïve persons was low. Several HBHCT studies have reported similarly low levels of linkage to care (Medley et al., 2013; Parker et al., 2015; Plazy et al., 2016). In contrast, other studies have reported high (>60%) levels of linkage to care (Barnabas et al., 2014, 2016; Tumwebaze et al., 2012). As stated previously (Plazy et al., 2016), it is difficult to compare these studies as each used a different definition of linkage to care. Additionally, across these studies, populations varied in terms of age composition, setting, access to HIV care services, prior knowledge of HIV-positive status, and previous engagement in HIV care. It is also worth noting that whereas in some studies HIV-positive persons were offered routine referral only (Genberg et al., 2015; Medley et al., 2013; Parker et al., 2015), others included interventions to facilitate referral (Barnabas et al., 2016; Tumwebaze et al., 2012; van Rooyen et al., 2013). In general, linkage to care was lower in the former compared to the latter studies.

As reported previously (Ruzagira, Grosskurth, Kamali, & Baisley, 2017) individuals in our trial who received follow-up counselling in addition to referral (intervention arm) after HBHCT were more likely to link to HIV care services than those who were referred only (control arm). Follow-up counselling may improve care seeking behaviour by providing specific information on how to access services (Ware et al., 2016), enhancing personal motivation (Knight, Van Rooyen, Humphries, Barnabas, & Celum, 2015), and reducing psychosocial barriers that inhibit linkage to care (Knight et al., 2015).

Surprisingly, some of the factors found in other studies to facilitate or hinder linkage to care were not associated with HIV care uptake in our study. In particular, age, sex, socio-economic status, time to reach the HIV clinic or education level, seem not to have influenced HIV care uptake in the trial.

Although ART initiation was higher among older adults than their younger counterparts, the association was not statistically significant, possibly owing to the small number of persons who initiated ART. Compared to younger persons, older persons may find it easier to accept their HIV-positive status and may be more likely to have the social support and resources that facilitate access to care (Naik et al., 2015). In the case of our study, it is also possible that many younger persons did not initiate ART because they were ineligible under the guidelines that were in effect in Uganda at the time. Indeed, at the time of linkage to care, only 53.0% of persons aged 18–44 years had a CD4 count of ≤500 cells/mm^3^, the ART eligibility threshold (Uganda Ministry of Health, 2014), compared to 84.0% among those aged ≥45 years.

It is worth noting that while the overall proportions of trial volunteers who obtained CD4 counts and initiated ART were low, uptake of these services among those who linked to care was high. For instance 95.3% of those who linked and had eligible CD4 counts initiated ART. This suggests that the linkage step is the main bottleneck to uptake of HIV care services in this population.

A limitation of our study is that we collected limited individual-level information during the HBHCT component of the study, so do not have data on a number of factors that have been shown to predict HBHCT uptake. Similarly, the analysis of factors associated with linkage to HIV care and uptake of HIV care services was limited by the lack of data on baseline CD4 counts, health status, and other factors that have been shown to predict these outcomes. Our assumption that individuals who were lost to follow-up did not link to care may have resulted in an underestimation of linkage to care. This is unlikely however, since such individuals are likely to have stigma-related worries or be exposed to circumstances that are associated with reduced linkage to care (Naik et al., 2015). Finally, the relatively small sample size may have limited the power to detect associations between some potential risk factors and outcomes. A strength of our study is that linkage to care, ART initiation, and CD4 count receipt could be verified through clinical records. Hence it is unlikely that uptake of these services was overestimated.

In conclusion, we found a high HBHCT uptake but low linkage to care, CD4 count receipt, and ART initiation among those who tested HIV-positive. Surprisingly, in our study uptake of HIV care services was not associated with commonly reported predictors of care uptake. Our study does therefore not provide guidance for additional interventions to increase linkage to HIV care, other than the counselling intervention that was tested in the trial.
